# Comparison of methods for donor-derived cell-free DNA quantification in plasma and urine from solid organ transplant recipients

**DOI:** 10.3389/fgene.2023.1089830

**Published:** 2023-01-27

**Authors:** Nicholas Kueng, Séverine Arcioni, Fanny Sandberg, Christian Kuhn, Vanessa Banz, Carlo R. Largiadèr, Daniel Sidler, Ursula Amstutz

**Affiliations:** ^1^ Department of Clinical Chemistry, Inselspital, Bern University Hospital and University of Bern, Bern, Switzerland; ^2^ Graduate School for Cellular and Biomedical Sciences, University of Bern, Bern, Switzerland; ^3^ Division of Medical Genetics, Central Institute of Hospitals, Valais Hospital, Sion, Switzerland; ^4^ Department of Nephrology and Hypertension, Inselspital, Bern University Hospital and University of Bern, Bern, Switzerland; ^5^ Department of Visceral Surgery and Medicine, Inselspital, Bern University Hospital and University of Bern, Bern, Switzerland

**Keywords:** dd-cfDNA, ddPCR, biomarker, cell-free DNA, high-throughput sequencing, liver transplant, urine, kidney transplant

## Abstract

In allograft monitoring of solid organ transplant recipients, liquid biopsy has emerged as a novel approach using quantification of donor-derived cell-free DNA (dd-cfDNA) in plasma. Despite early clinical implementation and analytical validation of techniques, direct comparisons of dd-cfDNA quantification methods are lacking. Furthermore, data on dd-cfDNA in urine is scarce and high-throughput sequencing-based methods so far have not leveraged unique molecular identifiers (UMIs) for absolute dd-cfDNA quantification. Different dd-cfDNA quantification approaches were compared in urine and plasma of kidney and liver recipients: A) Droplet digital PCR (ddPCR) using allele-specific detection of seven common *HLA-DRB1* alleles and the Y chromosome; B) high-throughput sequencing (HTS) using a custom QIAseq DNA panel targeting 121 common polymorphisms; and C) a commercial dd-cfDNA quantification method (AlloSeq^®^ cfDNA, CareDx). Dd-cfDNA was quantified as %dd-cfDNA, and for ddPCR and HTS using UMIs additionally as donor copies. In addition, relative and absolute dd-cfDNA levels in urine and plasma were compared in clinically stable recipients. The HTS method presented here showed a strong correlation of the %dd-cfDNA with ddPCR (*R* ^2^ = 0.98) and AlloSeq^®^ cfDNA (*R* ^2^ = 0.99) displaying only minimal to no proportional bias. Absolute dd-cfDNA copies also correlated strongly (*τ* = 0.78) between HTS with UMI and ddPCR albeit with substantial proportional bias (slope: 0.25; 95%-CI: 0.19–0.26). Among 30 stable kidney transplant recipients, the median %dd-cfDNA in urine was 39.5% (interquartile range, IQR: 21.8–58.5%) with 36.6 copies/μmol urinary creatinine (IQR: 18.4–109) and 0.19% (IQR: 0.01–0.43%) with 5.0 copies/ml (IQR: 1.8–12.9) in plasma without any correlation between body fluids. The median %dd-cfDNA in plasma from eight stable liver recipients was 2.2% (IQR: 0.72–4.1%) with 120 copies/ml (IQR: 85.0–138) while the median dd-cfDNA copies/ml was below 0.1 in urine. This first head-to-head comparison of methods for absolute and relative quantification of dd-cfDNA in urine and plasma supports a method-independent %dd-cfDNA cutoff and indicates the suitability of the presented HTS method for absolute dd-cfDNA quantification using UMIs. To evaluate the utility of dd-cfDNA in urine for allograft surveillance, absolute levels instead of relative amounts will most likely be required given the extensive variability of %dd-cfDNA in stable kidney recipients.

## 1 Introduction

Solid organ transplantation is widely accepted as the best treatment for various end-stage organ diseases, improving quality of life and reducing mortality. Even though the rate of graft survival has steadily increased over the years in both the short and long term, graft-related pathologies such as antibody- or cell-mediated rejection, infections and drug-related toxicity remain major hurdles to overcome. Improvements have been made regarding diagnostic tools leading to an increased graft survival, however biopsies remain the gold standard for diagnosis of allograft dysfunction. The significant costs, potential complications and inter-observer interpretation variability in allograft biopsies highlight the need for additional biomarkers to detect or exclude acute rejection, ideally at a subclinical stage, and other causes of allograft dysfunctions in a cost-efficient and less invasive manner.

Liquid biopsy has emerged as a novel minimally invasive approach using quantification of cell-free DNA (cfDNA) originating from the allograft, so-called donor-derived cfDNA (dd-cfDNA), in plasma. Large, prospective, multicenter studies (Bloom et al., 2017; Schütz et al., 2017; Khush et al., 2019; Knight et al., 2019; Jang et al., 2021) show the validity of dd-cfDNA to detect acute allograft injury. While most investigations focused on the relative amount of dd-cfDNA among the total cfDNA (%dd-cfDNA), recent data (Oellerich et al., 2019; Whitlam et al., 2019; Schütz et al., 2020; Bunnapradist et al., 2021) including large clinical studies suggest a potentially improved diagnostic performance with absolute quantification, i.e., dd-cfDNA copies/ml. Factors influencing the amount of circulating recipient-derived cfDNA such as exercise (Breitbach et al., 2012) in the short term, and changes in dynamic immunosuppressive regimens (Schütz et al., 2020) as well as other diseases (Swarup and Rajeswari, 2007) in the long term, highlight the shortcomings of dd-cfDNA quantification based solely on relative amounts. Furthermore, the majority of research so far has focused on plasma as the source of dd-cfDNA and only few studies have investigated dd-cfDNA in urine. (Martuszewski et al., 2021). Limited results show potential for urinary dd-cfDNA with improved practicality as a biomarker especially for, but not limited to renal allograft injury. (Martuszewski et al., 2021).

Different methods have been used for the detection and quantification of total and dd-cfDNA, namely quantitative PCR, droplet digital PCR (ddPCR) and high-throughput sequencing (HTS), which all differ in terms of cost, turnaround time and infrastructure requirements. Depending on the techniques either specific HLA mismatches, sex differences or common single-nucleotide or copy number polymorphisms were used as molecular targets to differentiate donor- and recipient-derived cfDNA. While the first two approaches utilize readily available donor genotype information, and thus, do not require separate donor genotyping or derivation of donor genotypes, the use of common polymorphisms can be applied more broadly independent of the presence of mismatches at individual loci. In recent years, several clinically (Bloom et al., 2017; Sigdel et al., 2018) and analytically (Grskovic et al., 2016; Altuǧ et al., 2019) validated HTS-based methods have entered the market, which are increasingly used as a non-invasive graft surveillance tool and are reimbursed by Medicare (USA) since October 2017. With clinical adoption, organ-specific %dd-cfDNA cutoffs have been proposed. However, these cutoffs are based on data originating from measurements with different methods. Despite the rise of dd-cfDNA as a non-invasive allograft monitoring approach, studies directly comparing methods, molecular targets and in different body fluids are lacking.

Here we present a direct head-to-head comparison of different analytical methods for the absolute and relative quantification of dd-cfDNA in urine and plasma from kidney and liver transplant recipients. Given the potential benefit of simultaneous dd-cfDNA quantification both as relative and absolute amounts, we developed a novel HTS-based method that incorporates unique molecular identifiers (UMIs) and evaluated its potential for the quantification of dd-cfDNA copies. Furthermore, we also investigated relative and absolute levels in urine and plasma in stable solid organ recipients and the correlation between both body fluids.

## 2 Materials and methods

### 2.1 Patient recruitment and sample collection

Patients were recruited at the Inselspital (Bern University Hospital, Switzerland) between August 2019 and August 2020. The study was approved by the ethics committee of the Canton of Bern, Switzerland (2019–00730). From liver and kidney transplant recipients who provided informed consent, whole blood and urine samples were collected once at a routine follow-up appointment or at up to three time points within the first week after transplantation. For the method comparisons, samples both collected during the early post-transplantation phase and during follow-up were used, in order to represent a broad range of dd-cfDNA quantities, and no sample selection based on specific patient characteristics or classification of allograft function was performed. For the comparison of dd-cfDNA quantities in urine and plasma, kidney transplant recipients were categorized as stable based on normal renal biopsy results and, in absence of biopsy results, on low average serum creatinine levels. Liver transplant recipients were considered stable if total bilirubin, aspartate aminotransferase (ASAT) and alanine aminotransferase (ALAT) levels were within the normal range. In addition, anonymized plasma samples from 20 healthy volunteers without a history of organ transplantation, no recent blood transfusion or pregnancy were utilized.

Venous blood samples were collected in two 7.5 ml K3 EDTA blood collection tubes (Sarstedt, Nümbrecht, Germany) and urine was collected in a 50 ml LoBind^®^ tube (Eppendorf SE, Hamburg, Germany). The Cell-Free DNA Urine Preserve (Streck Inc., La Vista, NE, USA) was added to urine samples to prevent cfDNA degradation and release of cellular genomic DNA (gDNA). For all downstream sample processing or storage steps, LoBind^®^ tubes were used.

EDTA whole blood samples were centrifuged within 2 h of collection at 2,000 g for 15 min at room temperature (RT). The plasma was re-centrifuged at 3,800 g for 10 min at RT. The full plasma supernatant and up to 1 ml of buffy coat were stored at −20°C until DNA extraction. The urine was centrifuged at 3′000 g for 15 min and the supernatant was initially stored at −20°C or −80°C.

### 2.2 cfDNA and gDNA extraction

The plasma was thawed at 4°C and cfDNA was extracted with the QIAamp Circulating Nucleic Acid Kit (Qiagen, Hilden, Germany), according to the protocol provided by the manufacturer. The 5 ml extraction scheme was applied using an elution volume of 150 µL and a second elution step by reapplying the eluate to the elution column. The eluate was subsequently concentrated using Amicon^®^ Ultra-0.5 30 K Centrifugal Filter Devices (Merck, Darmstadt, Germany) according to the instructions provided by the manufacturer with a centrifugation time of 3.5 min.

The urine samples were thawed at RT and the cfDNA was isolated using the Quick-DNA Urine Kit (Zymo Research, Irvine, CA, USA). The extraction was performed according to the “Cell-free DNA only” protocol provided by the manufacturer with a prolonged incubation with proteinase K at 55°C for 60 min instead of 30 min to ensure optimized protein digestion and increased cfDNA yield. Elution was performed with 40 µL DNA Elution Buffer pre-heated to 65°C and with a re-elution step.

The eluate volume was determined with a 10–100 µL pipette (Eppendorf SE) and the DNA concentration was measured using the Qubit^®^ 1X dsDNA HS Assay Kit and Qubit^®^ 4 fluorometer (Thermo Fisher Scientific, Waltham, MA, USA). The isolated plasma-derived and urinary cfDNA was subsequently stored at −80°C until further analysis.

### 2.3 Spike-in cfDNA series

Two cfDNA spike-in series were prepared, each mixing cfDNA extracted from plasma of two healthy volunteers with cfDNA from the first volunteer being used as recipient-derived cfDNA and cfDNA from the second volunteer being used as dd-cfDNA. Each series was prepared by adding increasing volumes of cfDNA from the first volunteer to an initial mix of the two samples, thus creating a series of mixtures containing known, decreasing proportions of cfDNA from the second volunteer. Approximately equal fragment size distributions were ensured using the High Sensitivity DNA Kit with the 2100 Bioanalyzer system (Agilent, Santa Clara, CA, USA). The mixture with the highest percentage of spike-in cfDNA used for the linearity assessment of the HTS method was also measured with ddPCR. The fractional abundance obtained by ddPCR was then used for calculation of the theoretical percentage spike-in of all samples in the series based on the dilution factor used.

### 2.4 Dd-cfDNA quantification by ddPCR

#### 2.4.1 Assays and validation

For the detection and quantification of dd-cfDNA by ddPCR, assays with sequence-specific TaqMan hydrolysis probes were used targeting alleles of the *HLA-DRB1* gene (Zou et al., 2017). Of the eight previously described assays, the following seven were ordered from Bio-Rad Laboratories Inc., Hercules, CA, USA, with HEX or FAM fluorophores, based on the high frequencies of the targeted alleles in the Swiss population: HLA-DRB1*01 (dHsaEXD29156242), HLA-DRB1*03 (dHsaEXD93426015), HLA-DRB1*04 (dHsaEXD10134188), HLA-DRB1*07 (dHsaEXD74403961), HLA-DRB1*11 (dHsaEXD22943507, dHsaEXD80505107), HLA-DRB1*13 (dHsaEXD54774880) and HLA-DRB1*15 (dHsaEXD29044653). Of note, the assay for the HLA-DRB1*03 allele was also specific for the gene and allele HLA-DRB3*03, while the HLA-DRB1*15 assay was also specific for the HLA-DRB1*16 allele. In addition, an assay targeting the *AGO1* gene was also used with both HEX (dHsaCP2500349) and FAM (dHsaCP1000484) fluorophores for the quantification of haploid genomes present in a sample.

To quantify cfDNA from male donor organs in female recipients, two assays targeting the Y-chromosomal *SRY* gene were used. For one, primers (forward: 5’—TGT​CCT​ACA​GCT​TTG​TCC​AG—3’; reverse: 5’—CCA​CTT​ACC​GCC​CAT​CAA​C—3’) and a probe (5’—FAM-ACCGCAGCAACGGGACCGCT-BHQ-1—3’) were newly designed for amplification under identical conditions as the *DRB1* alleles with an amplicon length of 77 bp. Primers and probe were ordered from Microsynth AG, Balgach, Switzerland and combined to a 20x concentrated assay mix with a final concentration in the reaction mixture of 450 nM for both the forward and reverse primers and 250 nM for the probe. Additionally, the ddPCR assay (dHsaCP2500472) targeting the *SRY* gene from Bio-Rad Laboratories Inc. was used.

To perform limited assay validation in addition to the published data (Zou et al., 2017), homozygous reference DNA samples from the Consanguineous Reference Panel were ordered from the International Histocompatibility Working Group (Fred Hutchinson Cancer Research Center, Seattle, WA, USA). These DNA samples were first amplified using whole genome amplification of 100 ng DNA per sample with the REPLI-g Mini Kit (Qiagen) following the protocol provided by the manufacturer and subsequently digested with HindIII (Promega Corp., Madison, WI, US) with 5 U of enzyme, for 2 h at 37°C. The DNA was subsequently quantified and diluted to a concentration of about 10 ng/μL. The ddPCR assays were tested with the corresponding reference target *HLA-DRB1* allele and non-target reference samples to ensure the absence of unspecific amplification. Further, the same tests were performed with cfDNA extracted from plasma of HLA-typed healthy individuals.

#### 2.4.2 Experimental setup and measurement

Each ddPCR reaction simultaneously contained one donor-specific assay and either a recipient-specific assay or an assay targeting both donor and recipient, using probes with different fluorophores. For each cfDNA sample, whenever possible, two different donor/recipient/total cfDNA assay combinations were measured per sample as triplicates, ideally one combination targeting donor- and recipient-specific *HLA-DRB1* alleles, and one combination targeting *AGO1* and a donor-specific *HLA-DRB1* allele or *SRY*. In samples where no two different assay combinations were available, two triplicates of the same assay combination were measured.

For ddPCR analysis, the QX200 AutoDG Droplet Digital PCR System (Bio-Rad Laboratories Inc.) was used and performed according to the manual “ddPCR^®^ Copy Number Variation Assays, Validated” (#10033173 Ver C) with the previously published PCR protocol (Zou et al., 2017) with an annealing temperature at 55°C. Up to 25 ng cfDNA were used per reaction. The QX200 droplet reader (Bio-Rad Laboratories Inc.) with the two-color detection system set to FAM/HEX and in the rare event detection (RED) mode was used for data acquisition.

#### 2.4.3 DdPCR data analysis

The QuantaSoft software v1.7.4 (Bio-Rad Laboratories Inc.) was used to analyze the ddPCR data. The measured copies were adjusted for the proportion of non-amplifiable copies as described below. In addition, the number of copies for alleles present with two copies per genome was adjusted to a single copy per diploid genome. The mean recipient- and donor-derived cfDNA copies/ml of the triplicates were calculated and used to calculate the mean for both assay combinations. For assay combinations, where the target was present in both the recipient and donor such as *AGO1* or matching *HLA-DRB1* alleles, the donor or recipient copy numbers were only measured indirectly and were determined by subtraction. Specifically, the copies of the target, which was only present in the recipient or donor, were subtracted from the copies of the target present in both the recipient and donor simultaneously, considering the zygosity of the targets.

#### 2.4.4 Adjusting for non-amplifiable fragments and concentration of urine

To correct for the proportion of target cfDNA fragments that were not PCR-amplifiable because they did not contain both PCR primers of the used assay, the formula (Equation 1) proposed by Oellerich et al. (Oellerich et al., 2019) was used. Instead of a sample-specific mean fragment length, a body fluid-specific fragment length was used (186 bp for plasma, 208 bp for urine), which was determined as described in the supplementary methods (Supplementary Presentation S1).

$$adjusted\;copy\;number\;\left[\frac{copies}{ml}\right]=copy\;number\;\left[\frac{copies}{ml}\right]\times \frac{\bar{fragment\;length}\;\left[bp\right]}{\bar{fragment\;length}\;\left[bp\right]-amplicon\;length\;\left[bp\right]}$$

Equation 1: Adjusting for the proportion of non-amplifiable fragments. Variables include the mean fragment length and the assay-specific amplicon length.

For measurements in urine, dd-cfDNA copies/ml were also normalized by the urinary creatinine (UCr) level at the time (±1.5 h) of urine collection because the concentration of urine can vary depending on the patient’s fluid intake.

### 2.5 Dd-cfDNA quantification by QIAseq HTS

#### 2.5.1 Assay design and validation

For the detection and quantification of dd-cfDNA with HTS, a custom QIAseq Targeted DNA Panel (Qiagen) was used, comprised of 121 common, disease-unrelated SNPs chosen based on their high population MAF (Supplementary Data Sheet S1). A total of 115 SNPs included in the panel were distributed over all 22 autosomes with an additional three SNPs for each the X and the Y chromosome. To maximize coverage for all targets considering the fragmentation of cfDNA, two specific primers (one on each strand) were designed for each target with the support of the QIAGEN bioinformatics team. The QIAseq protocol incorporates unique molecular identifiers (UMI) during library preparation, enabling the elimination of PCR amplification bias relevant in absolute quantification and low abundance variant detection with HTS.

#### 2.5.2 Library preparation and sequencing

Libraries were prepared using 10–40 ng cfDNA and sequenced according to the manual of the QIAseq Targeted DNA kit with 18 universal PCR cycles and using the QIAseq 96-Unique Dual Index Set A (Qiagen) to prevent index cross-talk. The library concentration was determined with the Qubit™ dsDNA 1x HS Assay Kit (Thermo Fisher Scientific) and the libraries were stored at −20°C until pooling. The sequencing pool was combined on the day of sequencing at a concentration of 4 nM using a mean library fragment size of 315 bp and subsequently stored at 4°C until denaturation. The pool was paired-end sequenced on an Illumina NextSeq 500 (Illumina, San Diego, CA, USA) with a NextSeq 500/550 Mid Output Kit v2.5 (300 Cycles). To generate 3-5 reads/UMI, about 6 million reads were sequenced per sample.

#### 2.5.3 Bioinformatic analysis

Demultiplexing was executed using the bcl2fastq Conversion Software v1.8.4. The resulting FASTQ files were imported into the CLC Genomic Workbench (v20) (Qiagen). Read mapping to the human reference genome GRCh37 and low-frequency variant calling was performed using a customized workflow (described in more detail in Supplementary Presentation S1) designed with the CLC Genomic Workbench, based on the “Identify QIAseq DNA Somatic Variants (Illumina)” workflow. For samples at or above 25% dd-cfDNA (as determined by ddPCR), the allele fraction for SNPs heterozygous in the recipient is expected to deviate significantly from 50%, and thus, the range of 25–75% used to identify such SNPs (Supplementary Presentation S1) could not be used. In such samples, a matching plasma or urine sample from the same patient with a %dd-cfDNA below 20% was used, if available, or a recipient gDNA sample obtained from buffy coat was sequenced to identify SNPs homozygous in the recipient and calculate the %dd-cfDNA. The Variant Call Format (VCF) files generated using the described workflow were used to subsequently calculate the %dd-cfDNA and absolute number of dd-cfDNA copies as described in Supplementary Presentation S1. Downstream data analysis was performed using Python v3.9.

The Python scikit-learn library v0.22.2 (Cournapeau et al., 2011), implementing the expectation-maximization (EM) algorithm (*n_components = 3*), was used to categorize SNPs classified as homozygous in the recipient into three clusters based on their %dd-cfDNA and to calculate the mean %dd-cfDNA and weight by cluster. With these means and weights, the mean sample %dd-cfDNA using the informative SNPs was calculated (Equation 2). A sample-specific noise factor, as the mean %dd-cfDNA calculated from the SNPs homozygous for the same allele in both the donor and recipient, was subtracted from the sample %dd-cfDNA.

$$\%dd–cfDNA=2\times \bar{{\%dd–cfDNA}_{hetero}}\times {weight}_{hetero}+\bar{{\%dd–cfDNA}_{homo,\;donor}}\times {weight}_{homo,\;donor}-\bar{{\%dd–cfDNA}_{homo,\;both}}\times {weight}_{homo,both}$$

Equation 2: Calculation of sample mean %dd-cfDNA using the EM algorithm results. The subscripts hetero and homo stand for heterozygous and homozygous. The subscript homo, donor stands for the SNPs homozygous in only the donor and homo, both stands for the SNPs that are homozygous in both the recipient and donor.

The mean absolute donor copies were calculated by averaging the donor copies of the SNPs in the homozygous group for the donor divided by two and the donor copies of the SNPs in the heterozygous SNP group.

### 2.6 Dd-cfDNA quantification by AlloSeq^®^ cfDNA

Using the AlloSeq^®^ cfDNA (RUO) kit (CareDx, Brisbane, CA, USA), libraries of 24 cfDNA samples were prepared simultaneously with an input of 10 ng and sequenced on a MiSeq instrument (Illumina) employing v3 chemistry with 150 cycles according to the kit manufacturer’s manual (IFU084, v3.1, 10.2020). The FASTQ files were analyzed and the %dd-cfDNA was calculated using the AlloSeq^®^ cfDNA Software (IFU085, v3.0, 07.2020). As the publicly available analytical validation of AlloSeq^®^ cfDNA (Grskovic et al., 2016) only focused on %dd-cfDNA values between 0.25% and 12% and to avoid additional recipient genotyping all samples analyzed in this study with AlloSeq^®^ cfDNA had a %dd-cfDNA around 20% or below as determined using ddPCR.

### 2.7 Statistical analysis

All statistical analyses were performed with the open-source programming language R for statistical computing (R Core Team, 2020) or Python v3.9. Data visualization was done with the R package *ggplot2* v3.3.5 (Wickham, 2016) and the Python library matplotlib v3.4.3 (Hunter, 2007). For correlation analysis the squared Pearson correlation coefficient as R^2^ and the Kendall’s Tau-a coefficient as *τ* were calculated. For regression analysis, the Passing Bablok and the linear least squares regression method were used. Passing Bablok regression was chosen as it is commonly used for method comparisons in diagnostic laboratories because it allows for imprecision in both of the compared methods as well as the inclusion of extreme values (Jensen and Kjelgaard-Hansen, 2006). The 95% confidence interval (95%-CI) for the Passing Bablok regression were calculated with the bootstrap (quantile) method. To test for heteroscedasticity the Goldfeld-Quandt test was used, implemented in the R package *lmtest* v0.9–39 (Zeileis and Hothorn, 2020). Bland-Altman, Q-Q and histogram plotting were performed using the R package *blandr* v0.5.1 (Edwards et al., 2017). Statistical testing between two continuous variables was performed with the Wilcoxon rank-sum test.

## 3 Results

### 3.1 Validation

**FIGURE 1:**
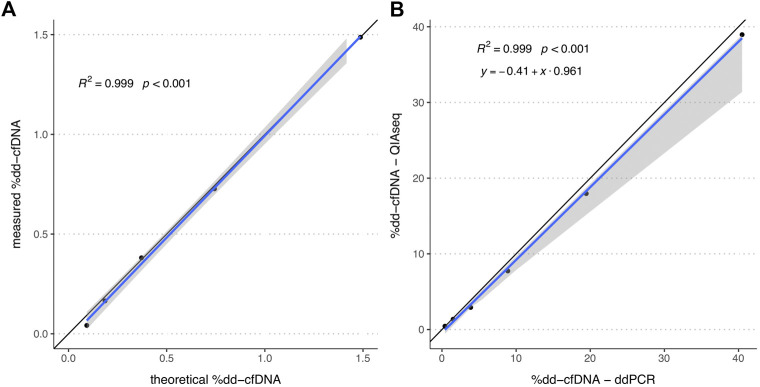
%dd-cfDNA of spike-in solutions measured by HTS alone and both HTS and ddPCR. **(A)** Scatter plot of the by QIAseq measured %dd-cfDNA *versus* theoretical %dd-cfDNA (*n* = 5). **(B)** Scatter plot of the %dd-cfDNA measured by ddPCR and QIAseq method (*n* = 6). The blue line represents the regression line calculated with the **(A)** linear regression method and **(B)** Passing Bablok regression method. The grey area represents the 95%-CI bounds calculated with the bootstrap (quantile) method.

To ensure that the HTS measurement results are directly proportional to the relative concentration of dd-cfDNA and exclude any potential saturation or noise effects, the linearity of the QIAseq HTS method was assessed. For that a spike-in series (*n* = 5) generated by mixing cfDNA from two unrelated healthy volunteers was measured using QIAseq. Linear regression analysis indicated a strong linearity (*R* ^2^ = 0.999, *p* < 0.001; Figure 1A) in the range of 0.1–1.5% which includes the %dd-cfDNA cutoffs used for most solid organ transplants (Edwards et al., 2017). An additional spike-in dilution series (*n* = 6) measured with QIAseq HTS and ddPCR with two different assay combinations showed a strong linear correlation (*R* ^2^ > 0.999, *p* < 0.001; Figure 1B) between both methods in the range of 0.5–40% without any systematic bias with an intercept at −0.41 (95%-CI: -1.03-0.16) but a small proportional bias (slope: 0.96; 95%-CI: 0.71–0.99). Furthermore, to establish the assay-specific background level, 20 plasma cfDNA samples from healthy volunteers were measured which showed a median %dd-cfDNA of 0.0075% (interquartile range, IQR: 0.000–0.037%).

For all samples measured in this study where the EM algorithm (see Methods 2.5.3) was applied, excluding one sample where the patient received an organ from his mother resulting in only heterozygous SNPs for the donor, the mean %dd-cfDNA was compared (*n* = 111) between the two groups of SNPs which were inferred to be heterozygous and homozygous, respectively, in the donor. A very similar %dd-cfDNA was observed in both groups (*R* ^2^ = 0.999, *p* < 0.001, Supplementary Figure S2). The median noise, calculated as the mean %dd-cfDNA from the SNPs inferred to be homozygous for the same allele in the donor and recipient, was not different from the noise observed in the healthy volunteer samples (*p* = 0.97) with 0.009% (IQR: 0.000–0.049%).

In the assessment of specificity of the *HLA-DRB1* ddPCR assays, no unspecific amplification of non-target alleles was observed in twelve homozygous carriers of different common alleles (DRB1*01:01, 03:01, 04:01, 04:04, 07:01, 08:01, 11:01, 11:04, 13:01, 13:02, 14:01, and 15:01). Additionally, the correlation between both assay combinations used to measure the total copies of a sample did not markedly differ whether both assay combinations measured the total copies directly (*R* ^2^ = 0.95, *p* < 0.001, *n* = 46), indirectly as the sum of donor and recipient copies (*R* ^2^ = 0.99, *p* < 0.001, *n* = 69) or one directly and the other indirectly (*R* ^2^ = 0.89, *p* < 0.001, *n* = 164) (Supplementary Figure S9).

### 3.2 Comparison between ddPCR and HTS (QIAseq)

To directly compare ddPCR to HTS (QIAseq), we assessed a total of 116 samples, of which 60 were collected early post-transplantation (<1 month) while the remaining 56 were collected at routine follow-up appointments. Of those, eight early post-transplantation plasma samples were excluded because SNPs, which had a %dd-cfDNA of zero in urine were >0% in plasma, suggesting the presence of third-party cfDNA, likely from a blood transfusion. An additional three plasma samples, all collected from the same patient post-transplantation, were excluded, as there was strong evidence that a blood transfusion introduced a substantial amount of third-party cfDNA, even though only plasma was available for analyses and the data could therefore not be compared to urine. The remaining 105 samples consisted of 38 plasma and 67 urine samples, of which 79 were collected from kidney, 24 from liver and two from a double transplant recipient (plasma and urine) who received both a kidney and liver from the same donor. Of those 105 samples, the donor cfDNA copies were indirectly measured with ddPCR (i.e., using subtraction of recipient copies from total copies; see Methods 2.4.3) in only two assay combinations and the recipient copies were indirectly determined in 84 assay combinations. Based on the ddPCR %dd-cfDNA (>29%) of six urine samples, cellular gDNA from the recipient was additionally sequenced for the calculation of the %dd-cfDNA (see Methods 2.5.3). For 19 urine samples (>18%) the matching plasma sample (<20%) from the same patient was used, while for four plasma samples from liver transplant recipients the matching urine sample (<8%) was used to identify SNPs with homozygous recipient genotype. Finally, for two post-transplantation samples, another sample from the same patient collected at a different time point with a %dd-cfDNA <20% was used.

After comparing the proportion of SNPs with zero %dd-cfDNA among all SNPs homozygous in the recipient for a sample to the corresponding sample %dd-cfDNA (Supplementary Figure S12), we chose 1% as the threshold, below which a random sampling effect was observable, i.e., no donor-specific sequences were observed for some SNPs even if a donor-specific allele was present due to random sampling error. Consequently, below this threshold, the %dd-cfDNA calculation based on the EM algorithm was expected to produce biased results due to this sampling error. Therefore, in all samples with a %dd-cfDNA <1%, the mean %dd-cfDNA over all homozygous recipient SNPs was considered, while above 1%, the %dd-cfDNA was calculated from the EM algorithm results.

**FIGURE 2:**
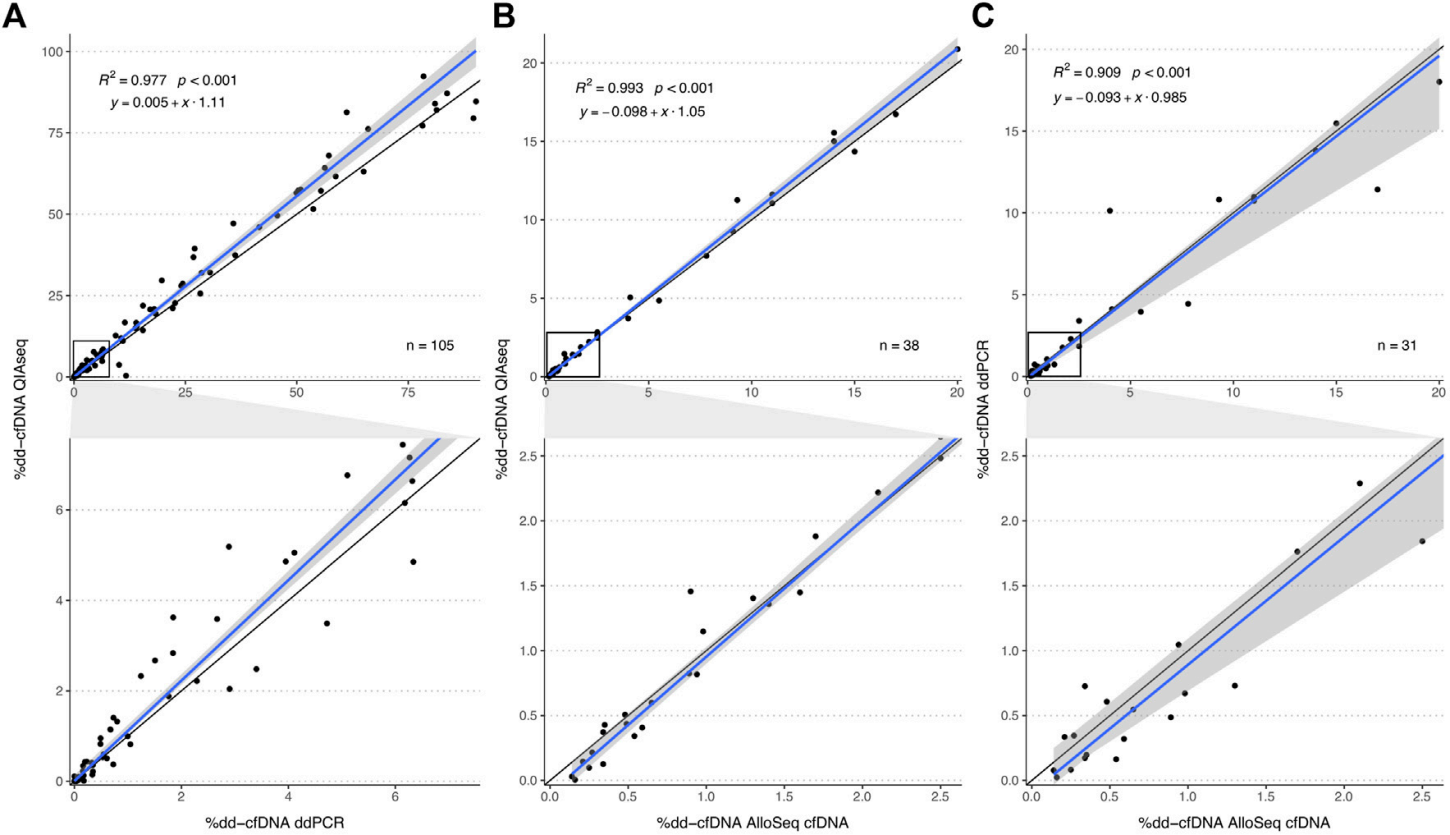
Method comparison plot for QIAseq, AlloSeq cfDNA and ddPCR. The plots in the second row visualize an enlargement of the area within the black squares in plots in the first row. Comparisons of the %dd-cfDNA between **(A)** QIAseq *versus* ddPCR, **(B)** QIAseq *versus* AlloSeq cfDNA and **(C)** ddPCR *versus* AlloSeq cfDNA. The blue line represents the regression line calculated with the Passing Bablok regression method. The grey area represents the 95%-CI bounds calculated with the bootstrap (quantile) method.

The %dd-cfDNA showed a strong correlation (*R* ^2^ = 0.977, *p* < 0.001, Figure 2A) between both methods with a slight bias towards a higher percentage measured with the HTS method and a significant heteroscedasticity (*p* < 0.001). Bland-Altmann plotting confirmed the bias (Supplementary Figure S6A); however, it was not significant below a %dd-cfDNA of 5%. The correlation was not markedly different when measurements were differentiated by body fluid (urine: *R* ^2^ = 0.988; plasma: *R* ^2^ = 0.969) or when differentiated by collection time point and allograft function (Supplementary Figure S13), whereas the proportional bias was only present for urine and not for plasma (Table 1).

**TABLE 1:** Passing Bablok regression parameters of the different method comparisons for the %dd-cfDNA and for specific sample subsets. The 95% confidence interval was calculated with the bootstrap (quantile) method.

		*intercept*			*slope*		
		estimate	95%-CI		estimate	95%-CI	
QIAseq versus ddPCR	overall	0.005	−0.032	0.109	1.11	1.05	1.15
	urine (*n* = 67)	0.147	0.000	0.783	1.12	1.04	1.16
	plasma (*n* = 38)	−0.018	−0.058	0.072	1.04	1.00	1.12
	follow-up (*n* = 56)	−0.016	−0.057	0.006	1.11	1.03	1.15
	post-transplantation (*n* = 49)	0.414	−0.020	0.805	1.08	1.02	1.18
QIAseq versus AlloSeq cfDNA	—	−0.098	−0.165	0.001	1.05	1.01	1.11
AlloSeq cfDNA versus ddPCR	—	−0.093	−0.196	0.112	0.99	0.81	1.05

**FIGURE 3:**
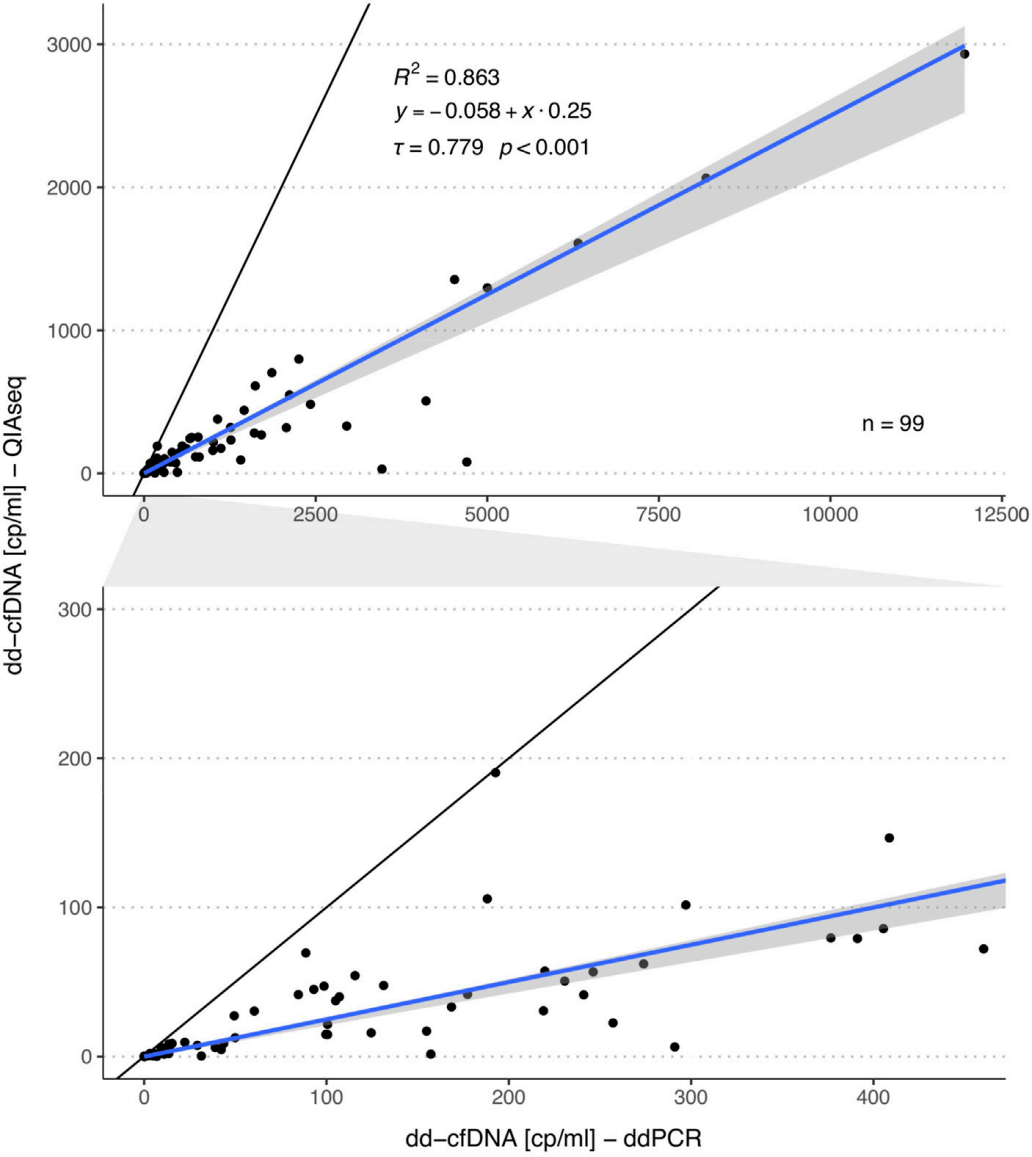
Comparison of the dd-cfDNA copy numbers measured by ddPCR and QIAseq method. The plot in the second row represents a zoomed area as outlined by the grey area in between both plots. The blue line represents the regression line calculated with the Passing Bablok regression method and the grey area the 95%-CI bounds calculated with the bootstrap (quantile) method.

For the same samples, we also compared the absolute number of dd-cfDNA copies between both methods. For this correlation analysis, we excluded six samples with >20,000 dd-cfDNA copies to reduce the influence of strong outliers on the analyses. A strong correlation was observed (*τ* = 0.779, *p* < 0.001, *R* ^2^ = 0.863, Figure 3). There was no systematic bias (intercept: -0.058; 95%-CI: -1.33-0.269), however, a much lower level of donor copies was measured with the QIAseq method (slope: 0.25; 95%-CI: 0.19–0.26).

### 3.3 Comparison to commercial kit

We further compared the QIAseq method with the commercially available AlloSeq^®^ cfDNA kit (CareDx) in a subset of 38 samples (13 urine and 25 plasma). We could show that both HTS methods strongly correlated (*R* ^2^ = 0.993, *p* < 0.001, Figure 2B) without any systemic bias, only minimal proportional bias (Table 1) and no significant overall bias between both methods (Bland-Altman plot, Supplementary Figure S6B). Similarly, for a subset of 31 samples measured both with AlloSeq^®^ cfDNA and ddPCR, there was a strong correlation (*R* ^2^ = 0.909, *p* < 0.001, Figure 2C) without any bias.

### 3.4 Dd-cfDNA levels in stable allograft recipients

**FIGURE 4:**
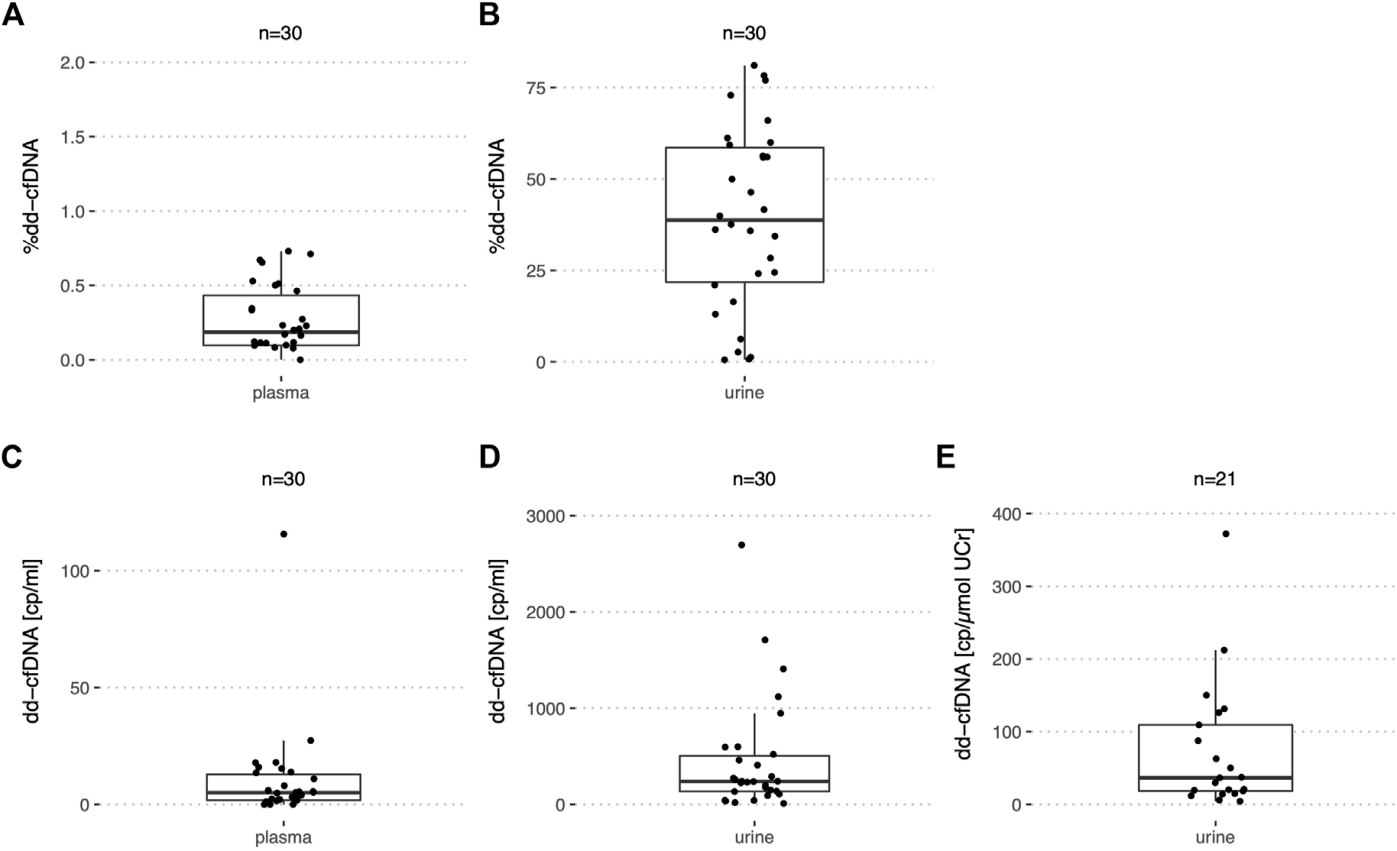
%dd-cfDNA and number of copies in plasma and urine from stable kidney transplant recipients. Boxplots of **(A,C)** the dd-cfDNA percentage and copies/ml in plasma; **(B,D)** dd-cfDNA percentage and copies/ml in urine and **(E)** dd-cfDNA copies normalized by UCr in urine.

Among a total of 30 urine samples analyzed by ddPCR from stable kidney recipients (Table 2) as supported by a negative biopsy result or low average serum creatinine, the median %dd-cfDNA was 39.5% (IQR: 21.8–58.5%) ranging from 0.54% to 81% (Figure 4B). The median dd-cfDNA copies/ml urine was 239 (IQR: 135–506) (Figure 4D) and 483 (IQR: 152–988) for recipient copies/ml. Urine creatinine levels at the time (±1.5 h) of urine collection were available for only 21 samples. When normalized by UCr [μmol/ml], the median donor copy number per μmol UCr was 36.6 (IQR: 18.4–109) (Figure 4E). The median recipient copy number was 80.2 (IQR: 24.3–620) copies/μmol UCr. A median of 2.6 ng cfDNA per ml (IQR: 1.5–5.6 ng/ml) were extracted from a median urine volume of 39.5 ml (IQR: 21.8–45.3 ml). For the stable kidney recipient urine samples with creatinine levels, the median total cfDNA concentration was 0.39 pg (IQR: 0.18–2 pg) per μmol UCr.

**TABLE 2:** Stable solid organ transplant patient demographics. DSA, donor-specific antibodies; PSC, primary sclerosis cholangitis; ADPKD, autosomal dominant polycystic kidney disease; post SOT-CKD, post solid organ transplantation chronic kidney disease.

Recipient Characteristics	Kidney (n =30)	Liver (n = 8)
Age, years		
mean (SD)	57 (13)	56 (16)
median	59	63
range	30–77	31–71
Gender		
male (%)	23 (76.7)	6 (75.0)
female (%)	7 (23.3)	2 (25.0)
Time post-transplant, days		
mean	1716	1289
median	939	355
DSA status		
DSA positive (%)	7 (23.3)	4 (50.0)
DSA negative (%)	23 (76.7)	3 (37.5)
unknown (%)	—	1 (12.5)
Indication for transplantation		
alcohol-related (%)	—	3 (37.5)
virus-related (%)	—	1 (12.5)
PSC (%)	—	2 (25.0)
glomerulonephritis (%)	5 (16.7)	—
cardiovascular (%)	5 (16.7)	—
ADPKD (%)	8 (26.7)	—
post SOT-CKD (%)	1 (3.33)	—
other (%)	11 (36.7)	2 (25.0)

In plasma of the same 30 stable kidney recipients, the median %dd-cfDNA was 0.19% (IQR: 0.01–0.43%) with a maximum of 0.73% (Figure 4A). The median dd-cfDNA copy number/ml plasma was at 5.0 (IQR: 1.8–12.9) (Figure 4C) and 2,320 (IQR: 1,733–3,347) for the recipient copies/ml. For the stable recipient plasma samples, a median of 13.0 ng cfDNA per ml (IQR: 8.2–17.5 ng/ml) was extracted from 4.0 ml (IQR: 3.5–4.2 ml). One outlier with increased levels of donor copies/ml compared to the other patients could each be observed in plasma and urine. However, the samples with increased levels in urine and plasma respectively, did not originate from the same recipient.

For the stable kidney transplant recipients, the interquartile range of the copies/ml plasma and copies/μmol UCr relative to the median was calculated. In plasma, this ratio was 3.2-fold greater for the dd-cfDNA copies compared to the recipient copies. In urine, the opposite was true with a factor three greater ratio for the recipient copies compared to the dd-cfDNA copies. The ratio for the dd-cfDNA was similar in plasma (2.22) and urine (2.48) but different for the recipient copies (plasma: 0.70; urine: 7.43).

**FIGURE 5:**
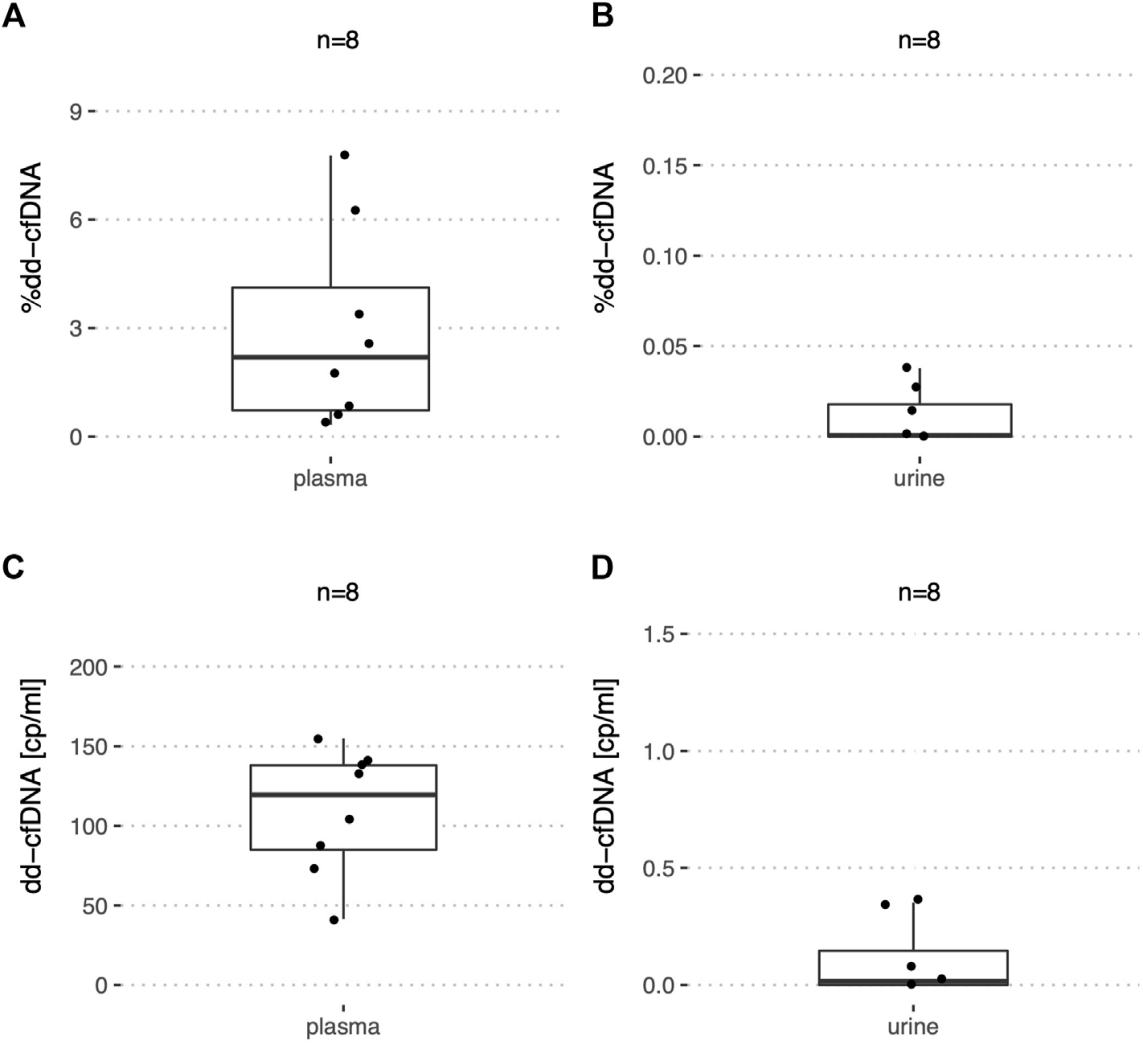
%dd-cfDNA and number of copies in plasma and urine from stable liver transplant recipients. Boxplots of the dd-cfDNA percentage and copies/ml for **(A,C)** plasma, and **(B,D)** urine.

In addition, urine and plasma of eight stable liver recipients (Table 2) were analyzed by ddPCR. With a median of 2.2% (IQR: 0.72–4.1%) (Figure 5A) in plasma, the %dd-cfDNA was higher than in plasma from stable kidney recipients (*p* < 0.001). The number of dd-cfDNA copies/ml was 120 (IQR: 85.0–138) (Figure 5C) and 4,918 (IQR: 2,165–14,434) for the recipient-derived cfDNA. A median of 25.2 ng cfDNA per ml (IQR: 10.8–41.5) was extracted from 4.3 ml (IQR: 4.0–4.6) of plasma.

In urine of liver recipients on the other hand, almost no dd-cfDNA copies could be detected with a median of 0.016 copies/ml and a maximum of 0.378 copies/ml (Figure 5D). The median of recipient-derived cfDNA copies was 1,737 copies/ml (IQR: 928–3,888). The %dd-cfDNA was measured at 0.0007% with a maximum at 0.0378% (Figure 5B), which was significantly lower (*p* < 0.001) compared to the kidney transplant patients. From urine, a median of 6.5 ng cfDNA per ml (IQR: 3.2–13.3) were extracted from 40.0 ml (IQR: 40.0–46.2).

There was a significantly higher (*p* < 0.05) concentration of total cfDNA in plasma from liver transplant recipients when compared to stable kidney recipients while the difference was not significant in urine.

In all 38 samples from stable solid organ transplant recipients, the donor copies were measured directly while for 66 assay combinations, the recipient copies were measured indirectly (see Methods 2.4.3).

**FIGURE 6:**
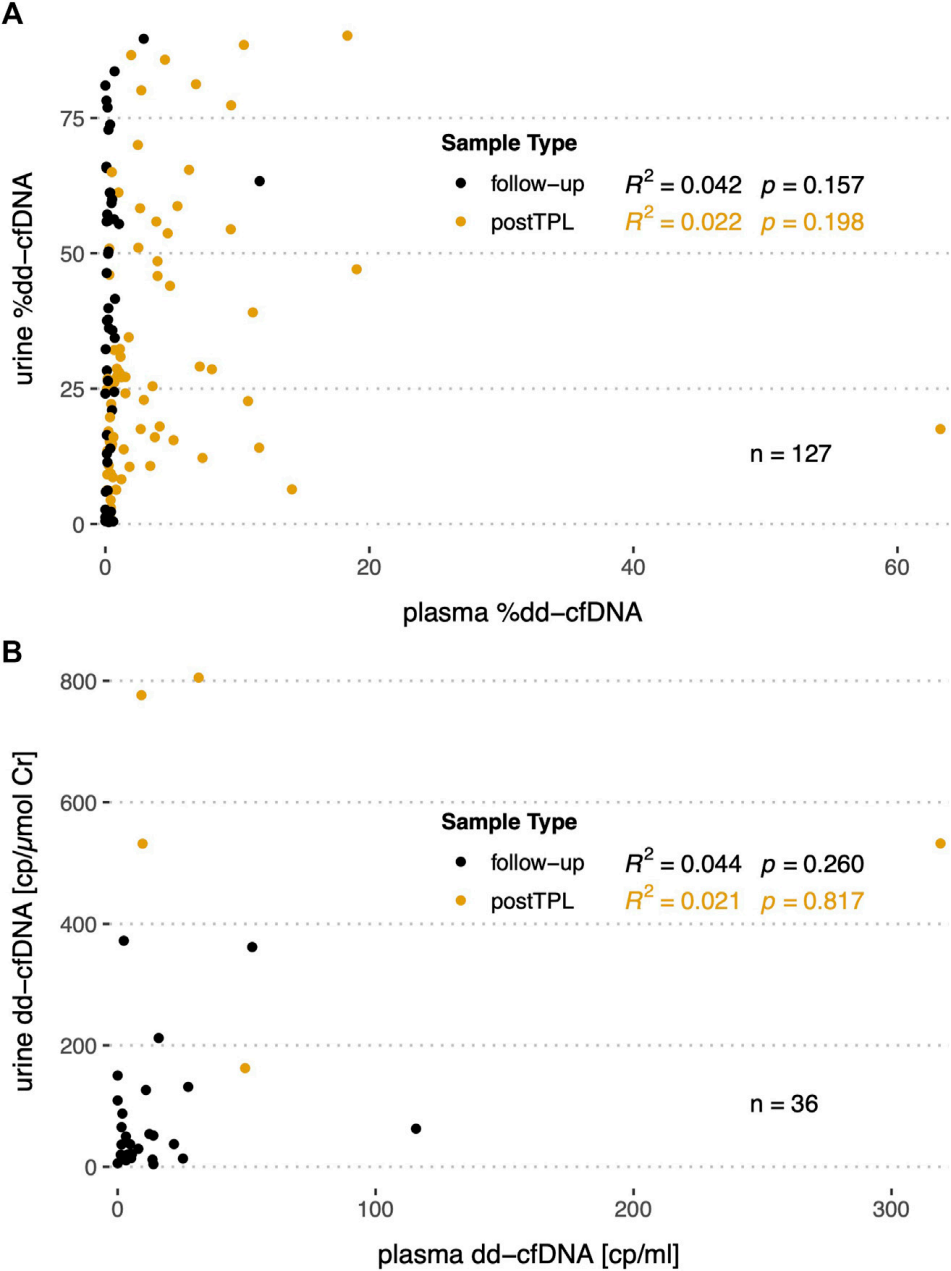
Correlation of the %dd-cfDNA and copy numbers in plasma *versus* urine in follow-up and post transplantation (postTPL) samples. **(A)** %dd-cfDNA correlation in plasma *versus* urine; **(B)** absolute dd-cfDNA copies/ml or copies/µmol UCr in plasma *versus* urine.

As for a set of patients %dd-cfDNA (*n* = 127) and absolute copies (*n* = 36) were measured in both urine and plasma, we assessed whether any correlation could be detected between the two body fluids. However, no significant correlation could be detected between urine and plasma for relative nor for absolute levels in both stable and early post-transplantation patients (Figure 6).

## 4 Discussion

With the emergence of dd-cfDNA as a novel minimally invasive biomarker for allograft rejection supported by numerous clinical validation studies, a variety of analytical methods for dd-cfDNA quantification have been developed and utilized. However, besides the analytic validation, studies directly comparing different quantification approaches are lacking. Here we showed that ddPCR and HTS, the two most commonly used methods for dd-cfDNA quantification, are highly comparable and display only minimal bias for %dd-cfDNA. Moreover, this study represents to our knowledge the first development of an HTS method using UMI to enable absolute quantification of dd-cfDNA copies. Finally, urine was shown to contain substantial amounts of dd-cfDNA in stable kidney allograft recipients, albeit with a highly variable fractional abundance, supporting the requirement for absolute quantification to unlock the potential of urinary-derived dd-cfDNA as a fully non-invasive biomarker for renal graft transplant surveillance.

This HTS method incorporating UMIs for dd-cfDNA quantification showed a high agreement of the %dd-cfDNA with ddPCR using assays targeting *HLA-DRB1* alleles. Due to the inclusion of early post-transplantation plasma samples and urinary cfDNA samples the range of %dd-cfDNA measured in this study was between 0% and >90%. This thus comprises the full range of %dd-cfDNA relevant in clinical diagnostics for monitoring of solid organ transplant recipients, including higher dd-cfDNA fractions relevant for analyses of urinary dd-cfDNA or of dd-cfDNA kinetics in the early post-transplantation phase. The suitability of the presented methods to quantify dd-cfDNA over a broad range is of great importance, as abnormal dd-cfDNA kinetics early post-transplantation, when high %dd-cfDNA are observed, might be indicative of early adverse events in kidney transplant recipients such as urinary tract infections, pre-renal acute kidney injury or surgical complications among others. (Gielis et al., 2018). Important to highlight is that these results show that even though both methods are based on completely different technologies and have different molecular targets, they result in a similar %dd-cfDNA. cfDNA release has been shown to be highly dependent on histone packaging and its susceptibility to DNase degradation, while also correlating with the transcription level of genes (Lo et al., 2021). As HTS methods interrogate multiple SNPs in the genome, any confounding effects due to different cfDNA fragmentation in different regions of the genome would likely be canceled out. For ddPCR, on the other hand, with the assays used here targeting only a single locus in the *HLA-DRB1* gene, such an effect is not expected. Moreover, even though the novel HTS method using UMI has not been as extensively validated as other HTS methods, we showed that a similar %dd-cfDNA could be measured when compared with AlloSeq^®^ cfDNA, an analytically and clinically validated HTS assay. It is also worth mentioning that even though AlloSeq^®^ cfDNA was validated for dd-cfDNA from plasma only, we also included urine samples in our analyses and still observed a high agreement between both methods. In contrast to the AlloSeq^®^ cfDNA bioinformatic analysis, which corrected for close donor-recipient relatedness using constants, we implemented an EM algorithm that adjusts automatically for any skewing in the SNP genotypes due to relatedness between donor and recipient. The strong concordance of the %dd-cfDNA from both donor genotypes among informative SNPs corroborates the robustness of the algorithm.

UMIs are commonly used for rare somatic variant detection in high-throughput sequencing of tumor DNA as they allow for PCR and sequencing error correction and the elimination of PCR duplicates. To our knowledge, this is the first published incorporation of UMI for the absolute quantification of dd-cfDNA using HTS. The strong correlation with ddPCR suggests that HTS with UMI can be used to further evaluate HTS-based absolute dd-cfDNA quantification. While the correlation with ddPCR was strong, a substantial proportional bias towards a lower number of dd-cfDNA copies detected by the HTS method was observed. This bias indicates that a lower proportion of input cfDNA molecules was sequenced with HTS compared to ddPCR-based quantification, which could have different causes. First, there could have been a significant loss of fragments during adapter ligation. Bead-based clean-up after adapter ligation might have further contributed to the loss of fragments. To establish a method-independent diagnostic cutoff for absolute dd-cfDNA copy numbers, it is necessary to evaluate whether a sample-independent correction factor could be used to adjust absolute dd-cfDNA quantities from different methods. The use of such a correction factor could be feasible given that the ligation efficiency and fragment loss during clean-up are expected to be similar for all samples.

We further applied ddPCR to determine and compare absolute and relative levels of dd-cfDNA in plasma and urine from stable liver and kidney transplant recipients. Plasma is the best-established source material for dd-cfDNA quantification so far and compared to urine, there is extensive literature on dd-cfDNA levels in plasma from allograft recipients. The median %dd-cfDNA observed here by ddPCR was similar to that reported by Bromberg et al. (Bromberg et al., 2017) using the assay from CareDx (median of 0.21%, IQR: 0.12%–0.39%) and Oellerich et al. (Oellerich et al., 2019) (median of 0.29%, IQR: 0.17%–0.56%) using a ddPCR-based method. Also for hepatic allograft recipients, the quantities observed here are comparable to the median %dd-cfDNA of 3.3% (95% CI 2.9%–3.7%) reported by Schütz et al. (Schütz et al., 2017) among stable liver transplant recipients.

While data on absolute dd-cfDNA copies in plasma from stable solid organ transplant recipients is limited, Whitlam et al. (Whitlam et al., 2019) reported comparable quantities (median of 7, IQR: 5–11) to those observed here. Dd-cfDNA copies in stable kidney recipients reported by Oellerich and colleagues were somewhat higher with a median of 25 copies/ml (IQR: 11–60), which could be attributed to an adjustment for extraction efficiency performed in that study. The similarity of the quantities observed here both for absolute and relative dd-cfDNA quantities compared to reports in the literature for stable recipients thus further supports the comparability of the different methods.

In contrast to plasma, urine would be a truly non-invasive source material with the additional benefit that it could be collected at home by the patients, and that it can easily be obtained in larger quantities compared to plasma. The %dd-cfDNA in urine from kidney recipients was substantially higher than in plasma and in a similar range as reported in small studies by Lee et al. (Lee et al., 2017) with a mean of 53.3% (IQR: 21–92%) (*n* = 8) analyzed by sex-mismatch ddPCR and Burnham et al. (Burnham et al., 2018) with a mean of 51.4% (*n* = 4) analyzed by HTS.

The results of the %dd-cfDNA in urine from stable kidney recipients revealed a large variability with values ranging from 0–80%, predominantly explained by the substantial fluctuations of recipient copies. This large variation in recipient copies may potentially mask relevant changes in the amount of dd-cfDNA in urine if expressed relative to the total copies. Considering these results, the quantification of absolute dd-cfDNA levels will likely be needed to further evaluate the potential of urinary dd-cfDNA for allograft monitoring.

With the comparison of urine and plasma for the same stable allograft recipients, it was observed that for patients who received a kidney, both the %dd-cfDNA and absolute copies were higher in urine compared to plasma. One might thus hypothesize that more DNA is released from the kidney into the urine than into the blood circulation. A difference in cfDNA release dynamics is also supported by the lack of correlation of relative and absolute levels of dd-cfDNA between plasma and urine from the same patients. However, before any conclusions can be drawn, the comparison of the total amount of cfDNA released into the blood and urine would be needed to confirm this.

With our analysis of urine samples from stable liver transplant recipients, we aimed to gain further understanding about transrenal dd-cfDNA, i.e., dd-cfDNA passing through the kidney into the urine. Even though a substantial number of dd-cfDNA copies could be detected in plasma in all liver recipients, interestingly, virtually no copies from the donor organ could be detected in urine from the same patients. These findings thus suggest very low levels of transrenal cfDNA in stable liver recipients. Of note, however, the extraction method used here for urinary-derived cfDNA had an isolation size range of 100–23,000 bp. Markus et al. (Markus et al., 2021) showed a substantially shorter fragment size of 81 bp for cfDNA fragments found in urine compared to plasma. It can therefore not be excluded that transrenal cfDNA predominantly consists of fragments significantly below 100 bp length, and thus may not have been detected in our study. Alternative extraction methods capable of extracting short fragments should be investigated for the further study of transrenal cfDNA in solid organ transplant recipients.

Finally, this study has some limitations. First, as samples used for the method comparison were not selected for specific allograft pathologies, this study could not be used to compare the diagnostic performance of the different dd-cfDNA quantification methods to identify rejections or other graft complications. While the strong correlations observed for %dd-cfDNA between assays makes a similar diagnostic performance likely, a comparison of absolute dd-cfDNA quantification methods with respect to their diagnostic performance should be performed in future studies. Further, a bit less than half of the 105 samples used for the ddPCR to HTS comparison were plasma samples. Therefore, body fluid-specific method agreement results are of limited statistical power. However, as both combined showed good agreement, big deviations are not expected if more samples were to be measured for each of the body fluids. A further limitation are the criteria, on which patients were deemed in a stable phase. For some kidney transplant recipients, a biopsy was not available and the decision was based on a low average serum creatinine. Similarly, the decision for the liver transplant patients was based on the levels of liver enzymes and total bilirubin, which show a low sensitivity and specificity in detecting subclinical acute cellular rejection. Since no liver biopsies were performed, no histological correlations could be shown. Therefore, it cannot be excluded that the data shown here also included patients with a subclinical rejection, which is associated with increased absolute and relative dd-cfDNA.

Overall, this first head-to-head comparison of the most commonly used approaches for the relative quantification of dd-cfDNA yielded highly comparable results with no or minimal biases. With the first incorporation of UMI in a dd-cfDNA HTS assay for dd-cfDNA quantification, we demonstrated the suitability of the presented custom HTS method for absolute dd-cfDNA quantification, even though the definition of a method-independent diagnostic cutoff for absolute dd-cfDNA may not be feasible given the substantially lower levels observed by HTS compared to ddPCR. Finally, high variability in recipient-derived cfDNA in urine indicates that methods enabling absolute dd-cfDNA quantification may be required to further evaluate urinary-derived dd-cfDNA for renal allograft surveillance.

## Data Availability

The counts per SNP and sample are available as Supplementary Material Data Sheet 2. The original raw data supporting the conclusions of this article will be made available by the authors upon reasonable request.

## Abbreviations

dd-cfDNA, donor-derived cell-free DNA; ddPCR, droplet digital PCR; EM, expectation-maximization; HTS, high-throughput sequencing; SNP, single-nucleotide polymorphisms; UCr, urinary creatinine; UMI, unique molecular identifiers.
