# Supplementary material for: Comparison of methods for donor-derived cell-free DNA quantification in plasma and urine from solid organ transplant recipients
Source: Front Genet. 2023 Jan 27;14:1089830. doi: 10.3389/fgene.2023.1089830 (PMC9916053; doi:10.3389/fgene.2023.1089830)
Supplement: Supplementary file 1 [file Presentation1.pdf]

Supplementary Methods

1. Estimation of body fluid-specific mean fragment length

To determine the body fluid-specific mean fragment length, the Kendall correlation between the %dd-cfDNA determined using two different assay combinations for a set of samples, measured with ddPCR, was iteratively calculated using mean fragment lengths in the copy number adjustment formula (Equation 1, Main Text) ranging from 110 to 10’000 bp in steps of two for plasma and urine separately (Figure S10/S11). For the iterations with plasma, a total of 160 samples were used with 32 from liver, 5 from double (same donor) and 126 from kidney transplant recipients. For urine, a total of 134 samples were used with 22 from liver, 2 from double (same donor) and 110 from kidney transplant recipients. The global maxima were manually defined resulting in a mean fragment length of 186 bp for plasma and 208 bp for urine.

2. High-Throughput Sequencing QIAseq Panel Bioinformatic Workflow

The resulting FASTQ files were imported into the CLC Genomic Workbench (v20) (Qiagen) with the options: «Remove failed reads», «Paired reads» and «Join reads from different lanes». Read mapping and variant calling was performed using a customized workflow based on the “Identify QIAseq DNA somatic variants” workflow provided within the CLC Biomedical Genomics Analysis plugin (Qiagen). As outlined in Figure S1, the basic workflow of this custom analysis pipeline started with the removal of the UMI and the annotation of the reads with the UMI information. The adapters with the sample indices were trimmed and the reads were mapped to the GRCh37 human reference genome. Afterwards, the UMI groups were generated by combining all reads with the same UMI into one group. UMI groups that only contained one read were assigned as singletons. “Similar” singleton groups were fuzzy merged (for further explanation, see “User manual for CLC Biomedical Genomics Plugin” v1.0). For each UMI group consisting of two or more reads, a single consensus read was created, called UMI read. With this step, instead of the whole group, only the UMI read was added to the previous location in the mapping, yielding mapped UMI reads as output.

In the next step, indels and structural variants were detected followed by local realignment improving the mapping of reads. With the Low Frequency Variant Detection tool, variant calling was performed based on the UMI reads. This combination allowed to detect variants with a low frequency (low %dd-cfDNA) and in samples with an unknown ploidy. Finally, variants thought to be false positives were removed based on a minimum variant frequency of 0.1% and forward/reverse balance of 0.05. This output was then stored as a VCF file (lfVCF). The output of the Low Frequency Variant Detection tool only contained loci, for which an alternate allele was detected but not the loci which were homozygous for the reference variant. In order to determine which and how many SNPs were homozygous for the reference allele, the “Identify Known Mutations from Sample Mappings” tool was used. With this tool, the allele count was determined, but without using any sequencing error estimation model. The output from this tool was also stored as a VCF file (mVCF).

For sequencing data from genomic DNA samples, the same workflow was used with the exception that only the "Identify Known Mutations from Sample Mappings" tool was used and no low frequency variant calling was performed, and the output was stored as a VCF file (gVCF).

Following the CLC Genomic Workbench workflow, further downstream analysis was performed using Python v3.9. As a next step, SNPs in both the lfVCF and mVCF, which were heterozygous in the recipient, were removed. In samples with an expected %ddcfDNA (based on ddPCR) below 20%, SNPs with an allele fraction in the range of 25 - 75% allele fraction were defined as heterozygous in the recipient. For samples ≥25% dd-cfDNA, the allele fraction for SNPs heterozygous in the recipient is expected to deviate from the 25 - 75% range. In these cases, the mVCF of the matching plasma or urine sample from the same patient was used if the %dd-cfDNA of the matching sample was below 20% (as described in the main text). In some cases, a sample in a time series showed a low %dd-cfDNA (<20%) and could be used as the mVCF for other samples with a higher %dd-cfDNA to identify the homozygous recipient SNPs. If no sample from the same patient with a %dd-cfDNA below 20% was available, the genomic information obtained from cellular DNA was used as a gVCF file. The SNPs from the gVCF file were also filtered for heterozygous SNPs with the same criteria as above, i.e. an allele fraction of 25 - 75%, and the output was a list of SNPs, which were considered homozygous in the recipient. With this information, SNPs from the lfVCF and mVCF files were removed if they were not in the gVCF/mVCF homozygous SNP list. This prevented retaining SNPs that appeared homozygous due to the high contribution of dd-cfDNA but were heterozygous in the recipient. Due to the intrinsic principle of variant calling, SNP loci for which only the reference allele was detected, were not included in the lfVCF. To identify SNPs, which were homozygous for the reference allele in both donor and recipient, target SNPs contained in the mVCF but not in the lfVCF were added to the latter file.

If not specified, using the genomic information from the recipient (gVCF or mVCF of another sample with low %dd-cfDNA), the count of UMI reads of the allele with the lower frequency among the two detected alleles for an individual SNP was used as donor copies and classified as dd-cfDNA. The %dd-cfDNA was then calculated as follows with the UMI counts from the lfVCF for each SNP:

$$\%dd\text{-}cfDNA = \frac{dd\text{-}cfDNA\ UMI}{dd\text{-}cfDNA\ UMI + recipient\text{-}derived\ UMI} = \frac{dd\text{-}cfDNA\ UMI}{total\ UMI}$$
