# Supplementary material for: Comparison of methods for donor-derived cell-free DNA quantification in plasma and urine from solid organ transplant recipients
Source: Front Genet. 2023 Jan 27;14:1089830. doi: 10.3389/fgene.2023.1089830 (PMC9916053; doi:10.3389/fgene.2023.1089830)
Supplement: Supplementary file 2 [file Presentation2.pdf]

Supplementary Figures

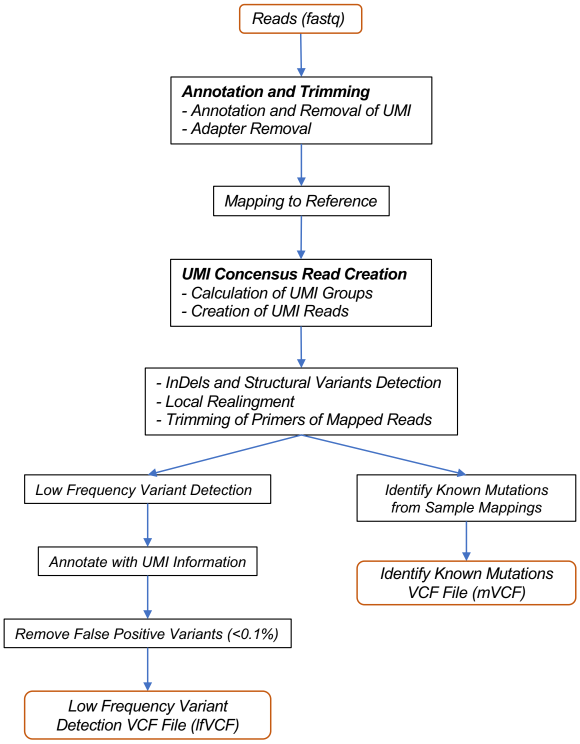

Figure S1: CLC Genomic Workbench Custom Workflow

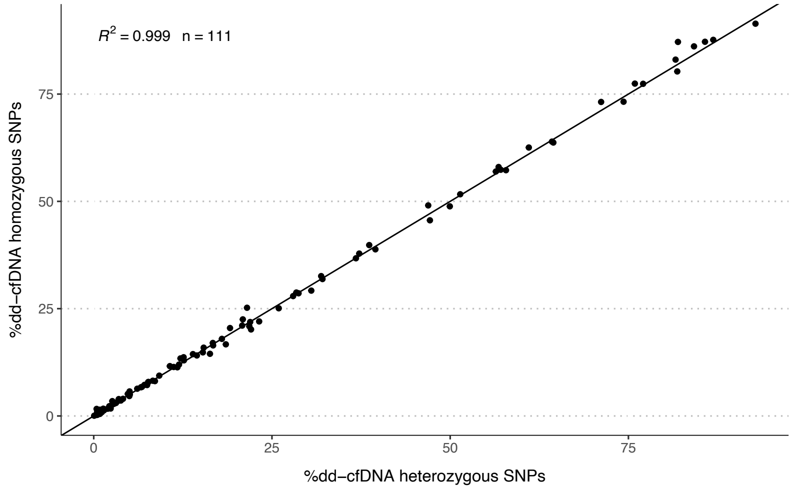

Figure S2: Comparison of the %dd-cfDNA calculated from the SNPs heterozygous in the donor versus homozygous in the donor. The black line represents the identity line.

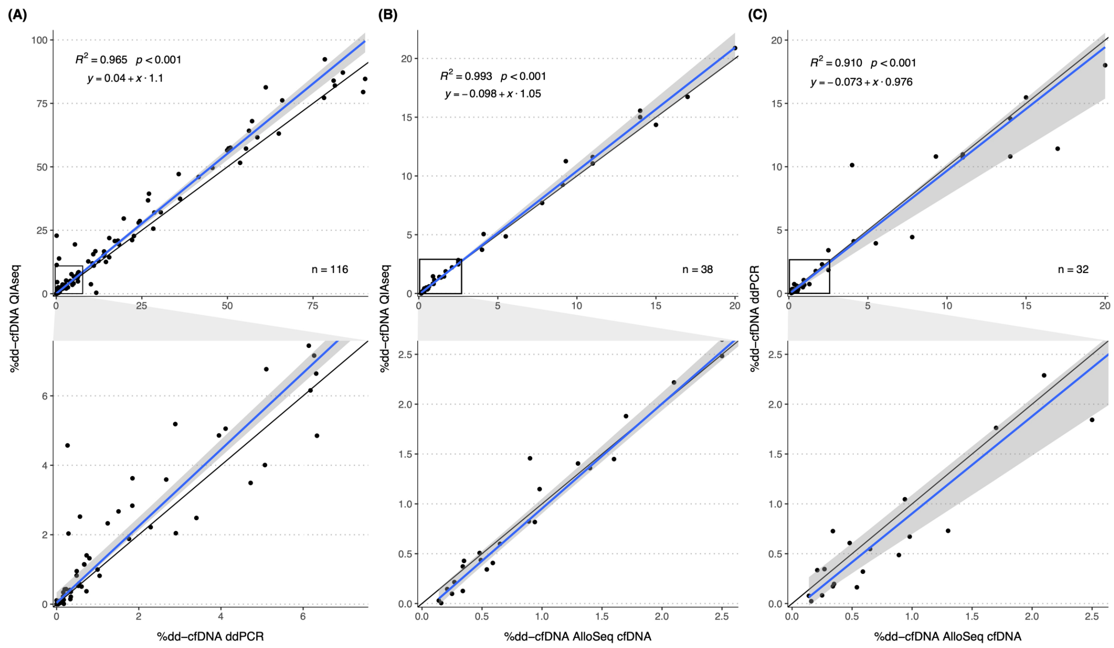

Figure S3: Method comparisons plots for the %dd-cfDNA between QIAseq, AlloSeq cfDNA and ddPCR (all samples). The plots in the second row visualize an enlargement of the area within the black squares in plots in the first row. Comparisons of the %dd-cfDNA between **(A)** QIAseq versus ddPCR, **(B)** QIAseq versus AlloSeq cfDNA and **(C)** ddPCR versus AlloSeq cfDNA. The blue line represents the regression line calculated with the Passing Bablok regression method. The grey area represents the 95%-CI bounds calculated with the bootstrap(quantile) method.

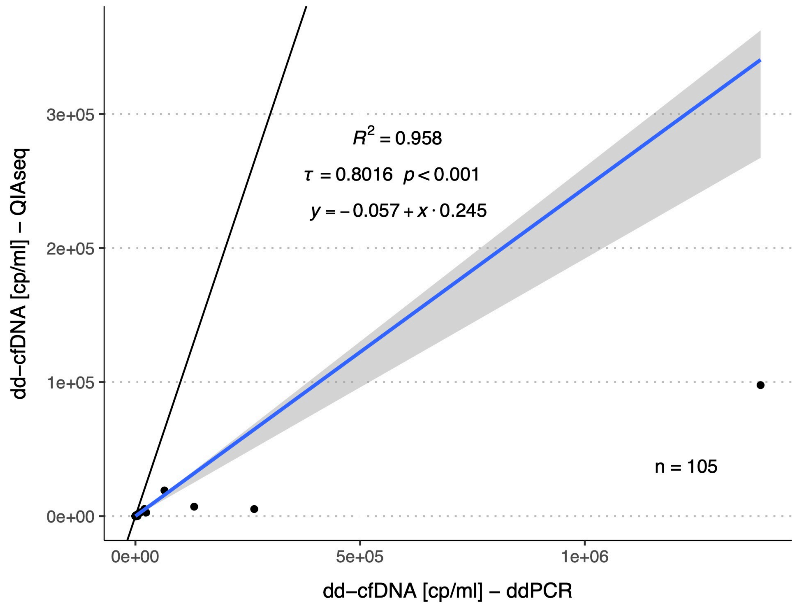

Figure S4: Comparison of the dd-cfDNA copy numbers measured by ddPCR and QIAseq method (all samples). The blue line represents the regression line calculated with the Passing Bablok regression method and the grey area the 95%-CI bounds calculated with the bootstrap(quantile) method.

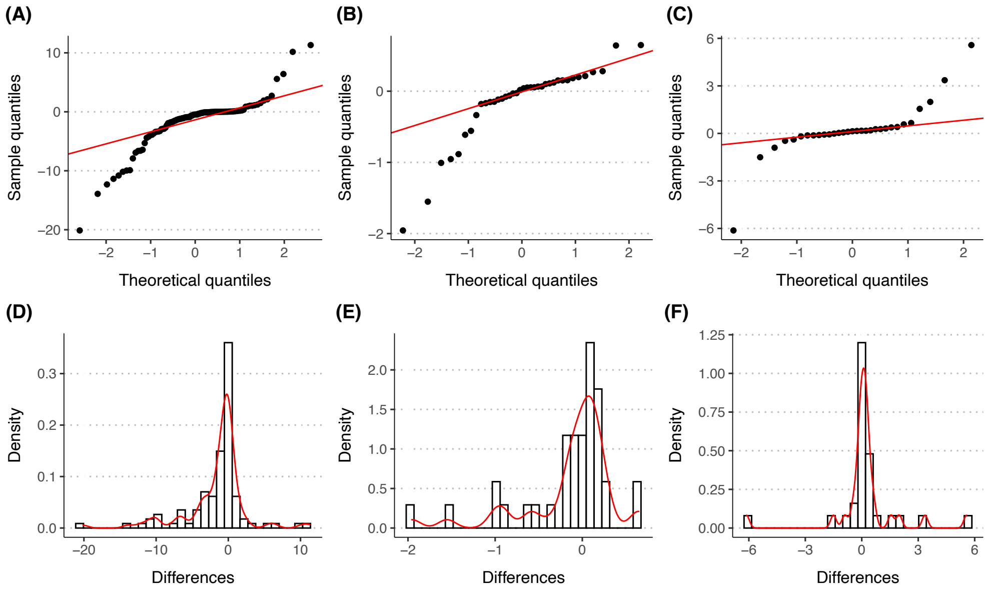

Figure S5: QQ plots and histograms for the method comparisons of the %dd-cfDNA. (A)-(C) represent QQ plots of the Bland-Altman differences and **(D)-(F)** histograms with density curves for the Bland-Altman differences for **(A)** and **(D)** QIAseq versus ddPCR (n = 105), **(B)** and **(E)** QIAseq versus AlloSeq cfDNA (n = 38) and **(C)** and **(F)** ddPCR versus AlloSeq cfDNA (n = 31).

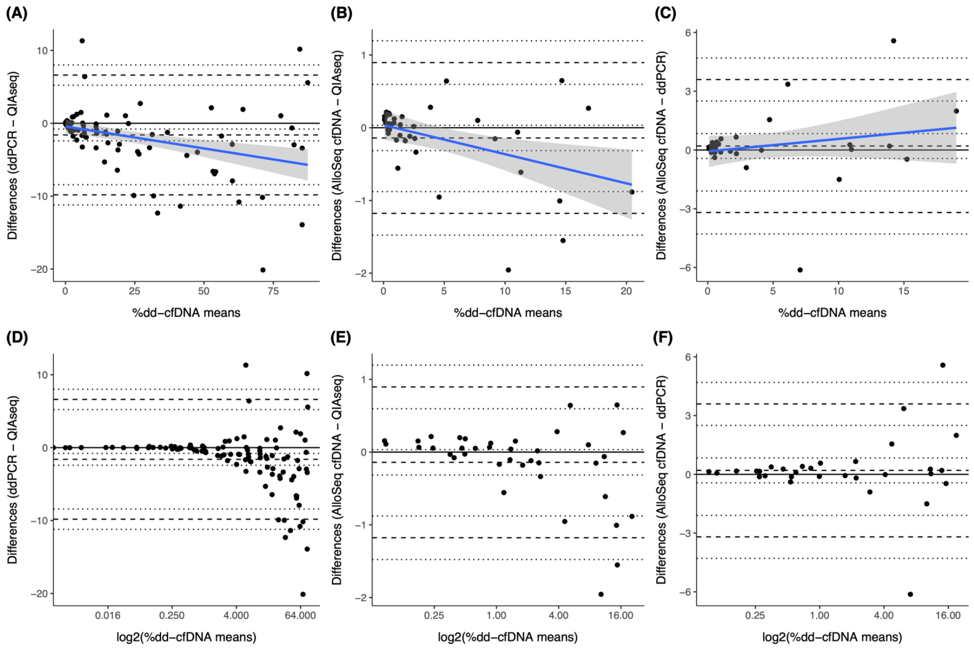

Figure S6: Bland-Altman plots for the method comparisons of the %dd-cfDNA. Bland-Altman plots for **(A)** and **(D)** QIAseq versus ddPCR (n = 105), **(B)** and **(E)** QIAseq versus AlloSeq cfDNA (n = 38) and **(C)** and **(F)** ddPCR versus AlloSeq cfDNA (n = 31). The x-axis scales of the plots in the second row are $\log_2$ transformed. The blue line in **(A)-(C)** represents the regression line and the grey area the 95%-CI bounds.

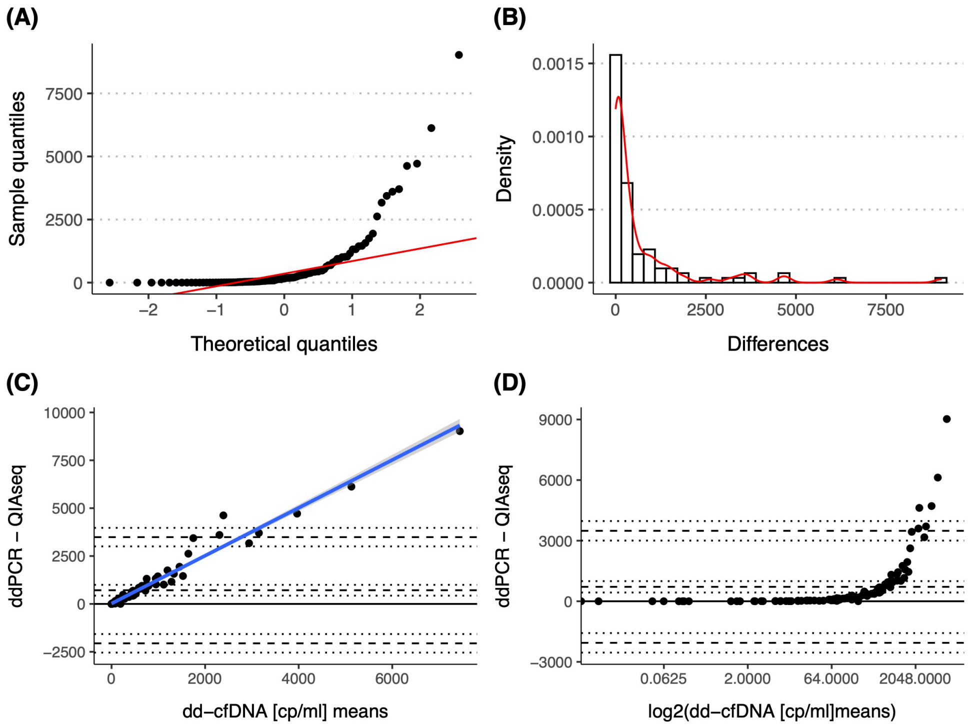

Figure S7: Distribution and Bland-Altman plots for the dd-cfDNA counts measured by QIAseq versus ddPCR. (A) Bland-Altman differences QQ plot, **(B)** histogram with density curve for the Bland-Altman differences, **(C)** Bland-Altman plot with the blue line as the regression line and the grey area as the 95%-CI bounds, and **(D)** Bland-Altman plot with $\log_2$ transformed scale on the x-axis. (n = 99)

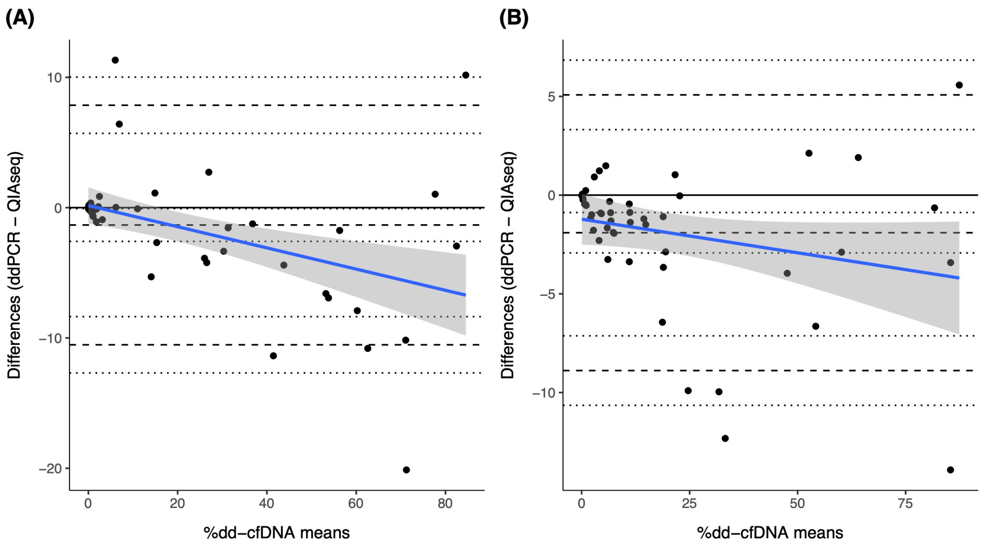

Figure S8: Bland-Altman analysis for the comparison of %dd-cfDNA between ddPCR and HTS. (A) Follow-up samples (n=56), **(B)** post-transplantation samples (< 1month) (n = 49). The blue line represents the regression line calculated with the Passing Bablok regression method and the grey area the 95%-CI bounds calculated with the bootstrap(quantile) method.

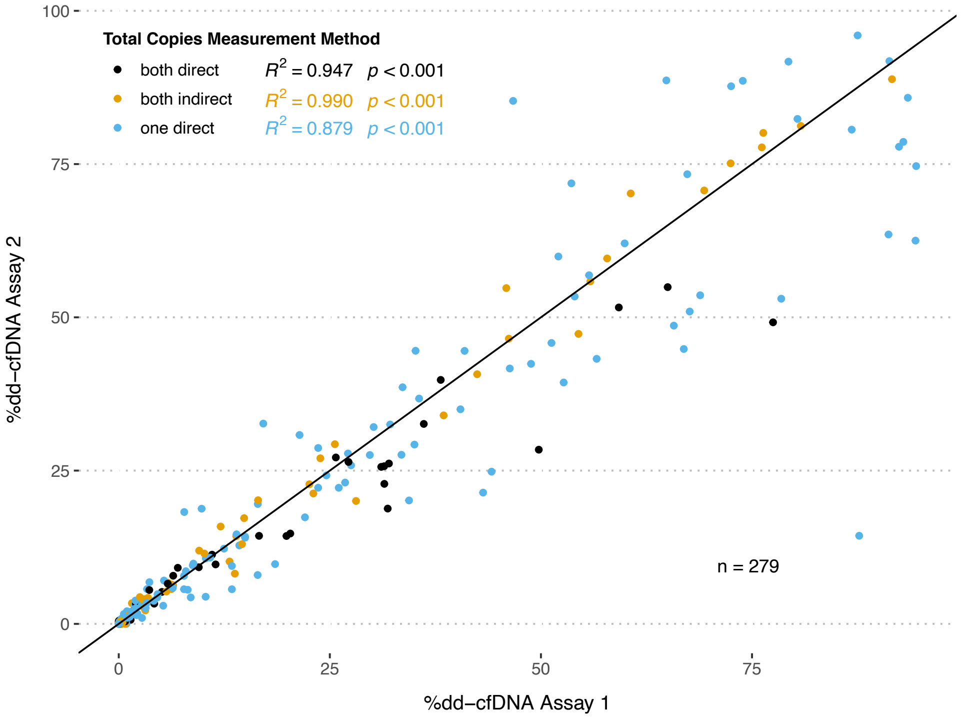

Figure S9: Correlation of the total copies between assay combinations according to the method. For this analysis, additional samples were included but the %dd-cfDNA was only measured with ddPCR. The black line represents the identity line.

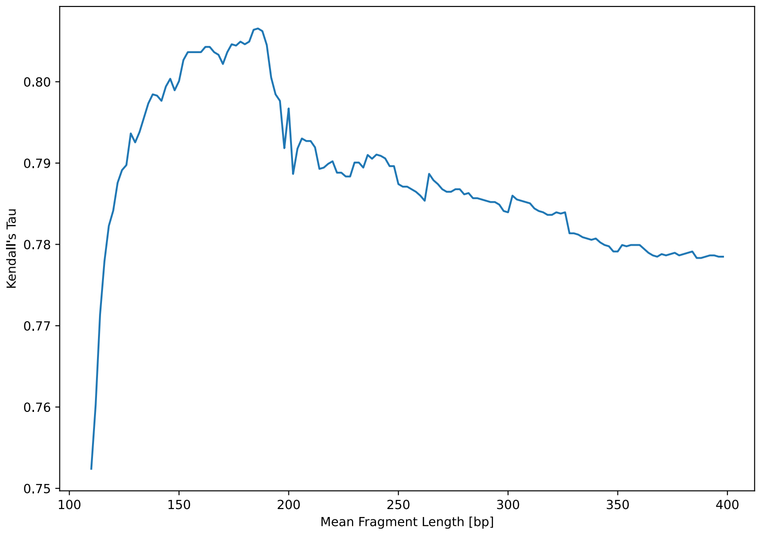

Figure S10: Kendall correlation of the %dd-cfDNA between both assay combinations for plasma samples. The mean fragment lengths used to adjust for non-amplifiable copies are on the x-axis while the Kendall's Tau resulting from the comparison of the %dd-cfDNA measured by two assay combinations in plasma samples (n = 160) is on the y-axis. The scale on the x-axis is limited to 400 bp even though the Kendall's Tau has been determined up to 10'000 bp.

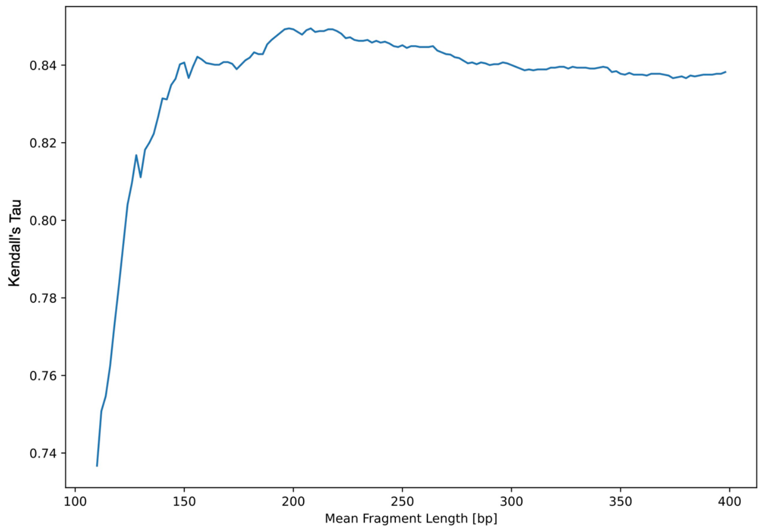

Figure S11: Kendall correlation of the %dd-cfDNA between both assay combinations for urine samples. The mean fragment lengths used to adjust for non-amplifiable copies are on the x-axis while the Kendall's Tau resulting from the comparison of the %dd-cfDNA measured by two assay combinations in urine samples (n = 134) is on the y-axis. The scale on the x-axis is limited to 400 bp even though the Kendall's Tau has been determined up to 10'000 bp.

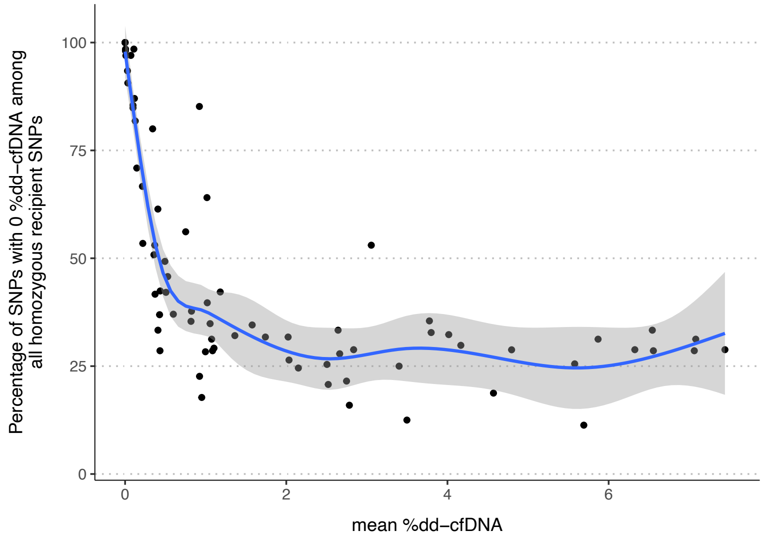

Figure S12: Relation between the proportion of homozygous recipient SNPs with 0% dd-cfDNA in a sample to its mean %dd-cfDNA over all SNPs. The blue line has been calculated using the generalized additive model (GAM) with the grey area as the 95%-CI bounds. (n = 73)

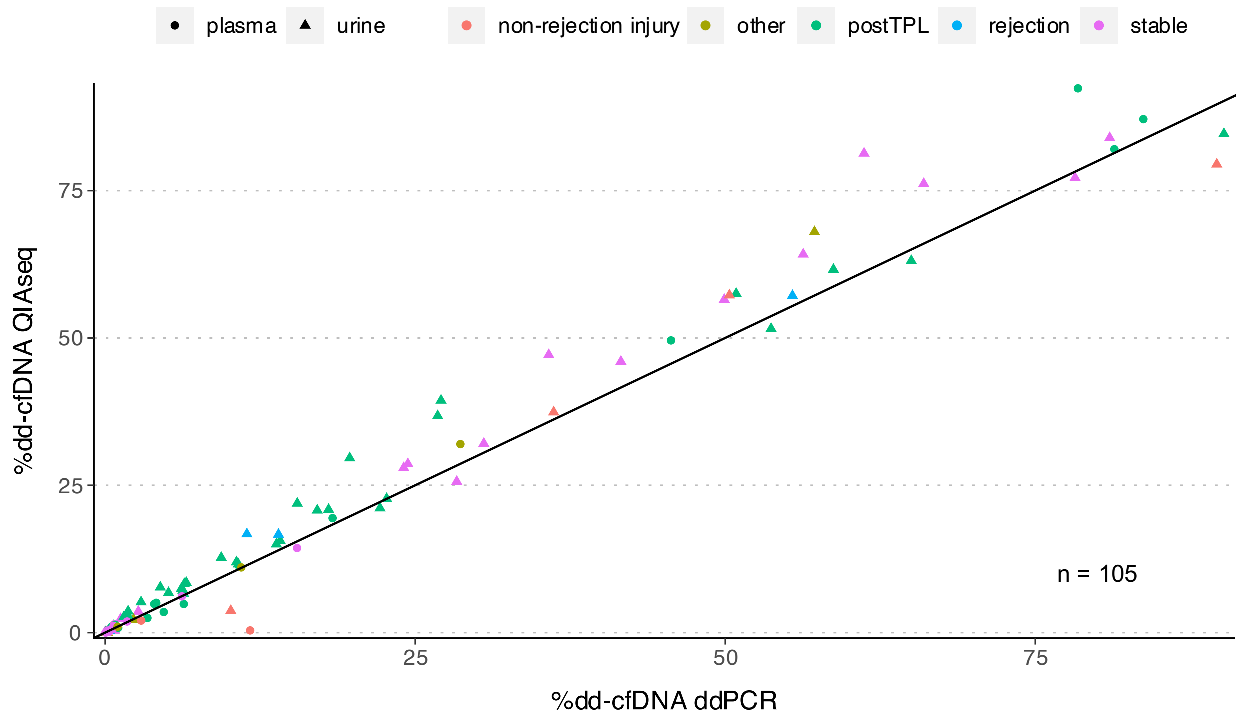

Figure S13: Method comparison plot for the %dd-cfDNA between QIAseq and ddPCR categorized by collection time and graft function. The samples in the non-rejection injury and rejection (antibody-mediated, T cell-mediated or borderline rejections) category were collected from patients with these diagnoses based on a biopsy performed within 30 days after sample collection. PostTPL stands for the post-transplantation phase which includes samples collected no later than 30 days after the transplantation. The category "other" includes all samples collected at a follow-up appointment from patients not deemed stable and without a biopsy-proven allograft injury. The black line represents the identity line.
